# Completeness of outcome description in studies for low back pain rehabilitation interventions: a survey of trials included in Cochrane reviews

**DOI:** 10.1186/1745-6215-16-S1-P24

**Published:** 2015-05-29

**Authors:** Greta Castellini, Silvia Gianola, Pamela Frigerio, Michela Agostini, Rosa Bolotta, Davide Corbetta, Monica Gasparini, Paolo Gozzer, Erica Guariento, Linda Li, Valentina Pecoraro, Valeria Sirtori, Andrea Turolla, Lorenzo Moja

**Affiliations:** 1 University of Milan, Milan, Italy; 2 Clinical Epidemiology Unit, I.R.C.C.S Galeazzi Orthopedic Institute, Milan, Italy; 3 Center of Biostatistics for Clinical Epidemiology, Department of Health Science, University of Milano-Bicocca, Monza, Italy; 4 Spinal Cord Unit, Niguarda Ca’ Granda Hospital, Milan, Italy; 5 Laboratory of Kinematics and Robotics. I.R.C.C.S. Fondazione Ospedale San Camillo, Venezia, Italy; 6 Service of Physiotherapy, National Institute of Injury Insurance, Milan, Italy; 7 Unit of Functional Recovery, Fondazione Centro San Raffaele del Monte Tabor, Milan, Italy; 8 Department of rehabilitation, Asl Biella, Italy; 9 PSS Tn, Villa Igea, Trento, Italy; 10 La Quiete casa di cura S.r.l, Varese, Italy; 11 Department of Physical Therapy, University of British Columbia, Vancouver, British Columbia, Canada; 12 Arthritis Research Centre of Canada, Vancouver, British Columbia, Canada; 13 Department of Biomedical Sciences for Health, University of Milan, Italy

## Background

Selection of appropriate outcome measures is crucial in clinical trials in order to minimize bias and allow for precise comparisons of effects between interventions [1-3].

## Objective

We aimed to assess the frequency and completeness of outcome measures in randomized controlled trials (RCTs) included in Cochrane systematic reviews (SRs), focusing on evaluations of the efficacy and safety of rehabilitation interventions for mechanical LBP.

## Materials and methods

**Figure 1 Checklist of completeness of outcome reporting:**
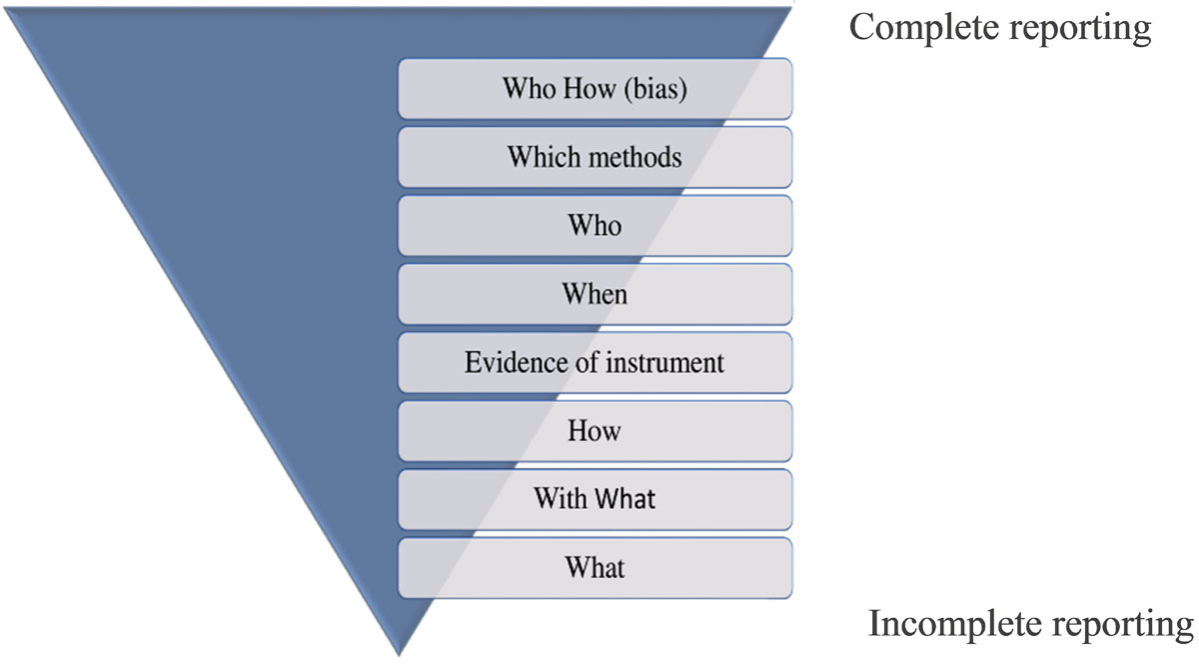
What: which outcome e.g., pain; With What: the instrument to measure that outcome e.g., visual analogue scale; How: how the instrument is applied e.g., visual analogue scale from 0 to 100; When: at which follow up e.g., immediately after the intervention period; Who: the assessor e.g., a physical therapist.; Who How: the detection status with reference to potential bias (i.e., systematic differences between groups in how outcomes are determined) e.g., blinding of the outcome assessor.

We performed a cross-sectional study of all RCTs included in all Cochrane SRs (full-text) published on The Cochrane Database of Systematic Reviews in February 2013. Two authors independently evaluated the type and frequency of each outcome measure reported in the full-text of RCTs, the methods used to measure outcomes, and the proportion of outcomes fully replicable based on the reported information (Figure 1).

## Results

**Figure 2:**
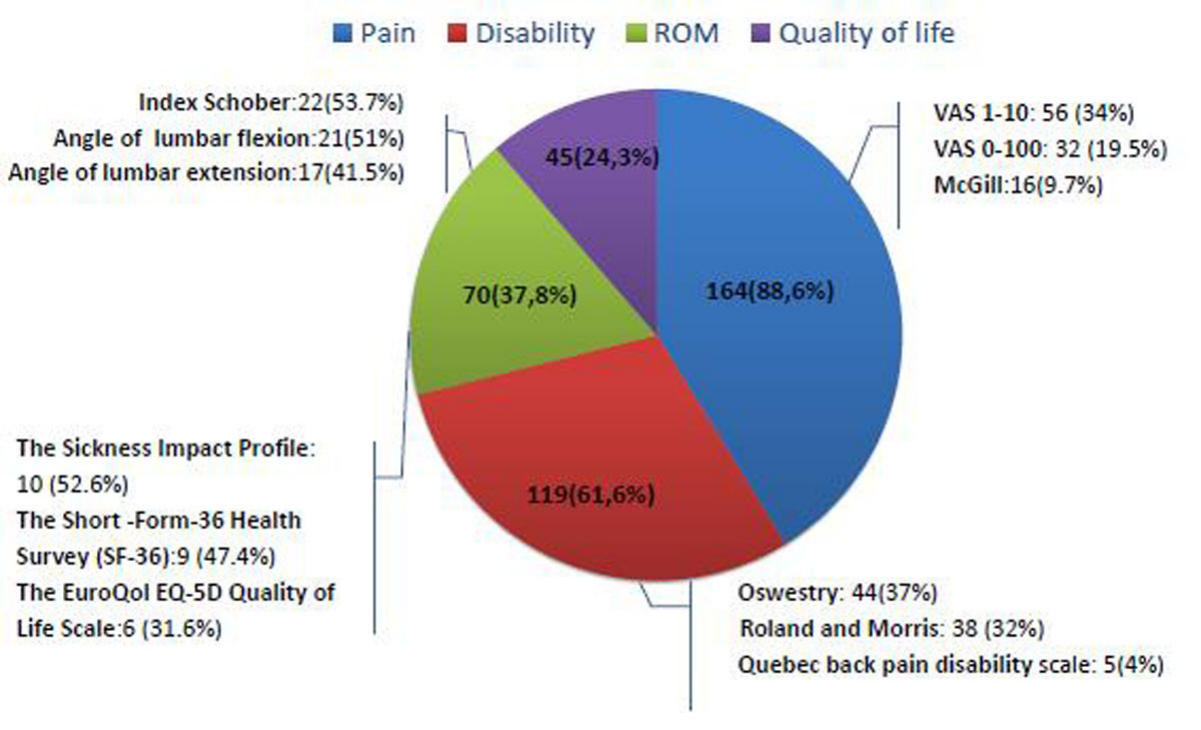
Top four outcomes for cumulative frequencies with relative top three measurement tools in our sample of 185 RCTs.

**Figure 3:**
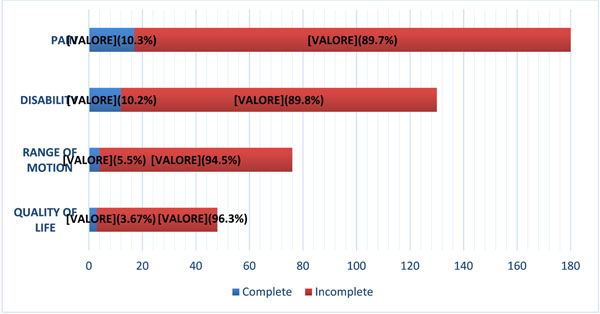
Completeness of outcome reporting in our sample of 185 RCTs.

Our literature search identified 11 Cochrane SRs, including 185 RCTs. Across all RCTs, thirty-six different outcomes were investigated. The outcomes most commonly reported were pain (165/185; 89,2%, 95% Confidence Interval (CI) 84.7% – 93.7 %), disability (118/185; 63,8%, 95% CI 56.9% – 70.7 %), range of motion (72/185; 38.9%95% CI 31.9% – 45.9%), and quality of life (45/185; 24,3%, 95% CI 18.1% – 30.5%) measured respectively by 70, 43, 41, 19 different measurement instruments (Figure 2). The procedure of blinding assessment was reported in 49.7% of the RCTs for pain (n= 82 RCTs) and 45% of RCTs for disability (n=53 RCTs). Pain, disability, range of motion, and quality of life outcomes were reported as fully replicable in 10.3% (n= 17 RCTs), 10.1% (n= 12 RCTs), 5.5% (n= 4 RCTs), and 6.6% (n= 3 RCTs) of the RCTs, respectively (Figure 3).

## Conclusions

A large number of outcome measures and a myriad of measurement instruments were used across all RCTs. The reporting was largely incomplete, suggesting better opportunities for the standardization of approaches and reporting.

## References

[B1] Coster WJ Making the best match: selecting outcome measures for clinical trials and outcome studies Am J Occup Ther 2013 67 2 162 170 10.5014/ajot.2013.006015 23433270 PMC3628620

[B2] Chapman JR Norvell DC Hermsmeyer JT Bransford RJ DeVine J McGirt MJ Lee MJ Evaluating common outcomes for measuring treatment success for chronic low back pain Spine 2011 36 21 Suppl S54 68 21952190 10.1097/BRS.0b013e31822ef74d

[B3] Deyo RA Battie M Beurskens AJ Bombardier C Croft P Koes B Malmivaara A Roland M Von Korff M Waddell G Outcome measures for low back pain research. A proposal for standardized use Spine 1998 23 18 2003 2013 (Phila Pa 1976) 10.1097/00007632-199809150-00018 9779535

